# Eating More Than We Need

**Published:** 1888-10

**Authors:** 


### EATING MORE THAN WE NEED.

Growth and waste and repair go on in a nearly uniform way the whole year through, but the amount of food necessary for these operations or purposes is surprisingly small. The generation of bodily heat requires a most variable quantity of food. In winter, with the temperature of the external air at zero, the temperature of the blood in healthy persons is 98.3 dgrees, and when the heat of summer drives the mercury of the thermometer near to or above that mark the blood still registers 98.3 degrees. The marvelous mechanism by which this uniform blood temperature is maintained at all seasons is not necessary to consider, but it must be evident to every one that the force needed to raise the temperature of the whole body to nearly one hundred degrees in winter is no longer needed in summer. The total amount of food needed for repair, for growth and for heating, physiology teaches us, is much less than is generally imagined, and it impresses us with the truth of the great surgeon Abernethy's saying, that "one-fourth of what we eat keeps us, the other three-fourths we keep at the peril of our lives." In winter we burn up the surplus food with a limited amount of extra exertion. In summer we get rid of it literally at some extra risk to health, and, of course, to life. We cannot burn it. Our vital furnaces are banked and we worry the most important working organs with the extra exertion of removing what would better never have been taken into the stomach.—*The Manufacturer and Builder.*

